# Mining tissue specificity, gene connectivity and disease association to reveal a set of genes that modify the action of disease causing genes

**DOI:** 10.1186/1756-0381-1-8

**Published:** 2008-09-19

**Authors:** Antonio Reverter, Aaron Ingham, Brian P Dalrymple

**Affiliations:** 1 Computational and Systems Biology, CSIRO Livestock Industries, Queensland Bioscience Precinct, 306 Carmody Road, St. Lucia, Brisbane, Queensland 4067, Australia

## Abstract

**Background:**

The tissue specificity of gene expression has been linked to a number of significant outcomes including level of expression, and differential rates of polymorphism, evolution and disease association. Recent studies have also shown the importance of exploring differential gene connectivity and sequence conservation in the identification of disease-associated genes. However, no study relates gene interactions with tissue specificity and disease association.

**Methods:**

We adopted an *a priori *approach making as few assumptions as possible to analyse the interplay among gene-gene interactions with tissue specificity and its subsequent likelihood of association with disease. We mined three large datasets comprising expression data drawn from massively parallel signature sequencing across 32 tissues, describing a set of 55,606 true positive interactions for 7,197 genes, and microarray expression results generated during the profiling of systemic inflammation, from which 126,543 interactions among 7,090 genes were reported.

**Results:**

Amongst the myriad of complex relationships identified between expression, disease, connectivity and tissue specificity, some interesting patterns emerged. These include elevated rates of expression and network connectivity in housekeeping and disease-associated tissue-specific genes. We found that disease-associated genes are more likely to show tissue specific expression and most frequently interact with other disease genes. Using the thresholds defined in these observations, we develop a guilt-by-association algorithm and discover a group of 112 non-disease annotated genes that predominantly interact with disease-associated genes, impacting on disease outcomes.

**Conclusion:**

We conclude that parameters such as tissue specificity and network connectivity can be used in combination to identify a group of genes, not previously confirmed as disease causing, that are involved in interactions with disease causing genes. Our guilt-by-association algorithm should be useful for the discovery of additional modifiers of genetic diseases, and more generally, for the ability to associate genes of unknown function to clusters of genes with defined functions allowing for novel biological inference that can be subsequently validated.

## Background

The understanding of the biology underlying phenotype is still a limiting factor in delivering the promise of high throughput genomics. However, as new datasets are available, new data mining methods are developed and the goal appears ever more achievable.

Among the high-throughput technologies, gene expression profiling has led to the identification of genes that perform in a coordinated manner allowing researchers to reasonably predict the role of genes for which no biological function was attributed, based on the known performance of other group members. These predictions rely on the guilt-by-association heuristic, widely invoked in genomics and with proven applicability [1].

At the same time, a comprehensive atlas of transcribed genes in humans has revealed that genes may be split into two broad categories based on the number of tissues they are expressed in [2]. Genes that are expressed in many tissues are designated as housekeeping (HK) while those that are expressed in few tissues are termed tissue-specific (TS).

Tissue specificity has subsequently been linked to a number of significant outcomes including level of expression [3], ability to detect cis-acting and trans-acting expression- quantitative trait loci [4], and differential rates of polymorphism [5], evolution [6] and disease-association [7]. In addition, we [8] and others [9,10] have demonstrated the importance of exploring differential gene connectivity in the identification of disease-associated genes using microarray gene expression data. More recently, the combination of text mining with gene interaction network analysis has been proposed to infer unknown gene-disease associations [11].

Furthermore, genes with a high degree of connectivity (network hubs) have been shown to be conserved across species [12] and their knockout phenotype more likely to be lethal [13]. Finally, based on sequence conservation across species, a computational algorithm has been developed to identify genes associated with disease [14]. However, no study relates gene interactions with tissue specificity and its subsequent likelihood of association with disease.

To address this situation, we mined three large independent datasets and classified transcribed human genes based on transcript abundance, tissue specificity, gene connectivity and disease association. We discuss how these factors relate to each other and, based on this new knowledge, implement a simple yet powerful guilt-by-association algorithm that allows us to identify several candidate genes that, while not previously associated with disease, may impact the development of diseases, including cancers, and hypothesize that many other members of this list will ultimately be confirmed as modifiers of various genetic diseases.

## Methods

### Data resources, edits and nomenclature

We merged three large datasets as follows: Firstly, we accessed expression data drawn from massively parallel signature sequencing (MPSS) covering 182,719 tag signatures across 32 tissues [2]. Tissues represented on the MPSS data included nine different central nervous system (CNS) areas (amygdale, caudate nucleus, cerebellum, corpus callosum, fetal brain, hypothalamus, thalamus, spinal cord, and pituitary gland) and 23 non-CNS organs (adrenal gland, bladder, bone marrow, heart, kidney, lung, mammary gland, pancreas, placenta, prostate, retina, salivary gland, small intestine, spleen, stomach, testis, thymus, thyroid, trachea, uterus, colon, monocytes and peripheral blood lymphocytes). A total of 18,677 unique genes were represented on the MPSS data and the number of expressed genes per tissue averaged 8,943 and ranged from 5,845 in pancreas to 12,267 in testis.

Secondly, we downloaded a set of 55,606 true positive interactions among 7,197 genes that were defined from functional studies [15]. This interactions dataset was built including 2,788 confirmed, direct, physical protein-protein interactions derived from the Biomolecular Interaction Network Database (BIND; ) [16], 18,176 confirmed human protein interactions from the Human Protein Reference Database (HPRD; ) [17], 22,012 direct functional interactions from the Kyoto Encyclopedia of Genes and Genomes (KEGG; ) [18], and 16,295 interactions derived from Reactome [19].

Finally, we used the microarray expression results generated during the profiling of systemic inflammation across 44,924 probe sets [20] and from which 126,543 interactions among 7,090 genes were reported [8]. The microarray experiment used 92 Affymetrix GeneChips (Affymetrix, Santa Clara, CA) to examine gene expression profiles in whole blood leukocytes immediately before and at 2, 4, 6, 9 and 24 h after intravenous administration of bacterial lipopolysaccharide (LPS) endotoxin to four healthy human subjects. For the control (placebo) data, four additional subjects were studied under identical conditions but without LPS administration.

For the present study, and to enable the merging of the three datasets, a number of edits were performed as follows: For the MPSS data, tags not expressed at more than 5 transcripts per million (tpm), in at least one tissue, were disregarded. The threshold of 5 tpm corresponds to the sensitivity of MPSS technology as claimed by the manufacturers and independently assessed in our laboratory [21]. Also, when the same gene was represented by more than one MPSS tag, the reading from the most abundant tag, summed across all tissues, was assigned to that gene. Finally, for the true positive interactions and the inflammation datasets, interactions involving genes not surveyed in the MPSS data were also discarded.

These criteria resulted in 15,050 genes [see Additional file 1] of which 5,198 and 4,950 were included in the true positive interactions and the inflammation datasets, respectively, and with 2,499 genes in common. In addition, a total of 6,151 (41%) of the genes were associated with disease according to OMIM database [22] as of September 19, 2007; and with 1,445 of them defined as disease-causing (i.e., associated with either known disease phenotype or polymorphic sequence known).

Hereafter, we refer to DIS to indicate the 6,151 genes from our resulting dataset that are disease-associated according to OMIM, and to NDIS to indicate the remaining 8,899 non-disease-associated genes also according to OMIM. Similarly, we refer to INT (and NINT) to indicate genes in our dataset for which interactions have (and have not) been reported.

### Data mining approaches

In order to further characterize the relationship existing between tissue specificity, gene connectivity and disease association, the 15,050 genes were classified as either TS or HK. To ensure that these two categories together represented the majority of the genes, we searched for category limits from either extreme of the distribution of the number of genes expressed in one, two, and up to 32 tissues, until equivalent categories were defined, cumulatively representing > 50% of the total number of genes. In doing so, there were 4,232 (28%) TS genes expressed in 1 to 4 tissues, and 4,006 (27%) HK genes expressed in more than 25 tissues. The remaining 6,812 (45%) genes were classified as non-specific (NS).

Finally, and in order to identify novel candidate genes impacting disease, we developed a guilt-by-association algorithm. Selection thresholds based on the average number of known interactions combined with the average proportion of DIS genes among their interactors were determined from DIS genes. These thresholds were then applied to genes in the NDIS category. Genes exceeding both thresholds were identified as likely disease-associated candidates.

## Results and discussion

### Initial gene groupings and unknown biological processes

**Figure 1:**
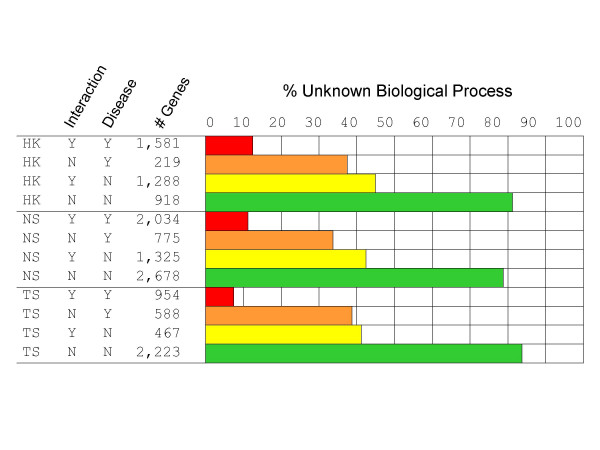
**Gene groupings**. Genes were classified as tissue-specific (TS), non-specific (NS) or housekeeping (HK). Among each class, the number of interacting and disease-associated genes is noted, and for each of the resulting 12 categories, the percentage of genes with unknown biological process ontology is given.

Figure 1 illustrates the way in which the 15,050 genes were simultaneously annotated as either disease-associated or included in the true positive interactions and the inflammation datasets. These genes were further classified as either TS, NS or HK, and the number of disease-associated and/or interacting genes contained within each of the resulting 12 categories was determined. The proportion of genes with unknown biological process was also registered.

As expected, the discovery of interactions as well as disease-association for a given gene provides additional biological knowledge, allowing inferences as to its genomic functionality. Nevertheless, the biological process of about 10% of these presumably well-characterized genes remains to be elucidated. On the other extreme, and highlighting the extent to which further research is needed, as many as 85% of NDIS, NINT genes and across the three expression categories (TS, NS and HK) belong to an unknown biological process.

### The impact of tissue-specificity

Among the myriad of complex relationships, some interesting patterns emerged. Consistent with previous findings [3], we observed a strong relationship between the number of tissues in which a gene was expressed and its level of expression (Table 1). Importantly, this relationship was unaffected by disease or interaction status.

**Table 1:** Relationship between the number of tissues in which a gene is expressed and a series of variables.

Variable	Correlation	Regression
Expression:		
Overall genes	0.706	2.034
Non-Interacting (NI) genes only	0.709	1.802
Interacting genes only	0.707	2.107
Non-Disease (ND)	0.707	1.769
Disease (D)	0.709	2.382
Non-Interacting and Non-Disease	0.691	1.759
Non-Interacting and Disease	0.764	2.039
Interacting and Non-Disease	0.719	1.803
Interacting and Disease	0.702	2.438

Proportion of interacting genes:		
Overall genes	0.949	0.012
Non-Disease genes only	0.942	0.013
Disease genes only	0.917	0.008

Proportion of disease genes:		
Overall	0.527	0.002
Non-Interacting genes only	-0.263	-0.001
Interacting genes only	-0.733	-0.004

Tissue specificity of interactors:		
Overall genes	0.887	0.112
Non-Disease genes only	0.736	0.062
Disease genes only	0.872	0.151

Proportion of disease genes among interactors:		
Overall genes	0.229	0.000
Non-Disease genes only	-0.048	0.000
Disease genes only	0.575	0.001

**Figure 2:**
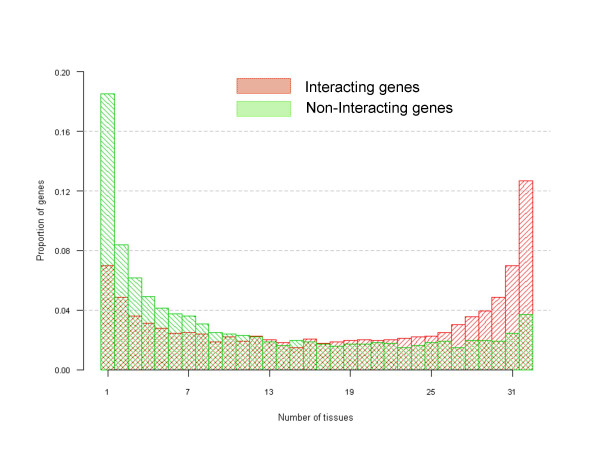
**Frequency histogram of gene expression**. For each gene, the tissues showing expression at more than 5 transcript per million were counted and the histogram explored separately for non-interacting (green) and interacting genes (red). The two distributions are statistically different (Kolmogorov-Smirnov test P-value < 0.001).

Overall, the distribution of the expression of genes among tissues was grossly bimodal. However, this bimodality vanished when the distribution was examined separately for INT and NINT genes (Figure 2). INT genes are over-represented among HK genes, while NINT genes are predominantly TS. We conclude that the more tissues a gene is expressed in, the higher its chances of interacting with at least one other gene, irrespective of the tissue-specificity of this second gene.

**Figure 3:**
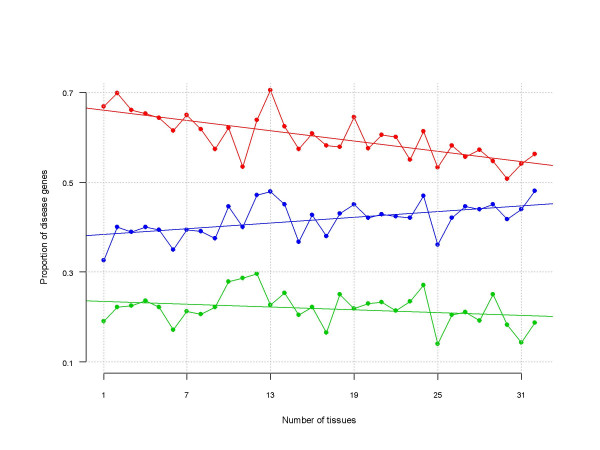
**Disease association and tissue specificity**. Relationship between tissue specificity (x-axis) and proportion of disease-associated genes (y-axis) computed using all genes (blue pattern), and separate for non-interacting (green pattern) and interacting genes (red pattern).

Figure 3 presents the relationship between tissue specificity and proportion of disease-associated genes. The overall Pearson correlation coefficient (PCC) was moderate (0.53) yet significant (P = 0.0019) indicating an increase in the number of DIS genes among broadly expressed genes. Computing the PCC conditional on interaction status results in a non-significant PCC of -0.26 (P = 0.1459) for NINT genes, and a strong negative PCC of -0.73 (P < 0.0001) for INT genes. This counterintuitive pattern of correlation is representative of the Simpson's Paradox [23] with the paradox being that, although INT genes tend to be expressed in many tissues, those that are expressed in a tissue specific manner are more likely to be DIS. This is likely due to the increased number of relationships an interacting HK gene would have compared to a TS equivalent, thereby increasing the likelihood of a mutation leading to a detrimental and potentially lethal outcome, as previously determined [6]. We conclude that it is not so much that TS genes are more likely to be associated with disease, but rather that HK genes associated with disease are rarely observed.

### Gene interactions in the context of tissue-specificity and disease association

Our analyses revealed that interacting HK genes are more likely to interact with genes that are also HK (PCC = 0.89; P < 0.0001) and vice-versa (i.e., TS genes are more likely to interact among themselves). Importantly, this correlation remained strong when conditioning on disease status (Table 1). Also, interactions between two HK genes were 12.8 times more frequent (P < 0.0001) and 3.3 times more cohesive (P < 0.0001) as measured by the clustering coefficient, than interactions between two TS genes. The clustering coefficient is a measure of network cohesiveness and captures how many neighbours of a given gene are connected to each other.

**Figure 4:**
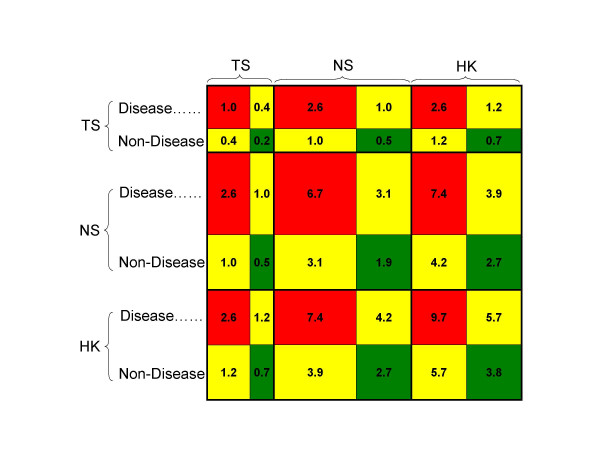
**Relating gene connectivity with disease association and tissue specificity**. Percentage of gene-gene interactions that exists between two groups of genes depending on their tissue specificity (TS: tissue-specific, NS: non-specific, and HK: housekeeping) and disease association. Colours indicate interactions between two disease-associated genes (red), between a disease-associated and a non-disease-associated gene (yellow), and between two non-disease-associated genes (green). The size of the rectangles indicates the relative number of interacting genes in each group.

Similarly, interactions between two DIS genes were 3.1 times more frequent (P < 0.0001) and 1.6 times more cohesive (P < 0.001) than interactions between two NDIS genes (Figure 4). Consistent with our results, genes associated with similar disorders have been shown to have higher likelihood of physical interactions between their products and a higher expression profiling similarity for their transcripts [24].

### Identification of candidate disease genes via guilt-by-association

Given our measurement confirming that like associates with like, we developed a guilt-by-association algorithm with the aim of identifying candidate genes among the previously classified non-disease subset. Our guilt-by-association algorithm starts by examining the connectivity properties of the DIS genes. In this context, DIS genes were found to be involved, on average, in 12 interactions (ranging from 0 to 176). Also on average, their interactors were themselves DIS genes in 75% of instances. Importantly, while only 1,132 (or 18.4%) of DIS genes had > 12 interactions (revealing the skewedness in the number of interactions), 651 (or 57.5%) of them interacted with DIS genes > 75% of the time. When these same thresholds (i.e., > 12 interactions and > 75% of DIS genes among interactors) were applied to NDIS genes, we revealed the presence of 112 genes [see Additional file 2], including 26 TS, 50 NS and 36 HK, that while not being associated with disease, have higher than average connectivity degree (> 12 connections) and higher than average proportion (> 75%) of genes in OMIM among their connectors. Table 2 presents the number of genes in the contingency table underlying our guilt-by-association algorithm.

**Table 2:** Contingency table underlying the guilt-by-association algorithm

Disease Associated?	Number of Connections	% Disease-associated genes among interactors	
		
		≤ 75	> 75
Yes	≤ 12	3,112	1,907
	> 12	481	651

No	≤ 12	7,853	705
	> 12	229	112

Number of disease- and non-disease-associated genes by thresholds on number of connections and percentage of disease-associated genes among interactors. The thresholds are obtained from exploring disease-associated genes and correspond to the average number of connections (12) among disease-associated genes and the average proportion of disease-associated genes (75%) among their interactors. The 112 non-disease-associated genes (bottom right cell) form the basis of the newly reported disease-associated genes [see Additional file 2].

To assess the optimality of our approach, we repeated the analyses using only the 1,445 DIS genes (out of the initial 6,151) with known disease phenotype and either sequence mutation or molecular basis known as those declared as truly disease-associated. The new thresholds for connectivity and proportion of DIS genes among interactors were 12 and 35%, respectively. The new list of candidate genes included 127 genes of which 107 were assessed as DIS in the initial list of 6,151. Assuming the remaining 20 genes are indeed false positives, this implies a precision of at least 84%.

It should be noted that precision alone is not enough to assess the goodness of a classifier, as it is only concerned with the ratio of identified genes that are positive, but not with the total number of discovered genes.

In order to further ascertain the optimality of various location parameters to be used as thresholds in the guilt-by-association algorithm, we explored the proportion of truly disease associated genes from the total number of captured genes and the results are presented in Table 3. While the median performs slightly better (i.e. by up to 1.03 times better, or 78.9 over 76.3) than the mean when used as a threshold for the proportion of disease genes among interactors, this improvement is at the expense of generating substantially larger lists of candidate genes. When exploring the number of connections, the mean is very close to the 75^th ^percentile, indicating the skewness in the connectivity distribution with most genes having few connections and few genes having many connections. Also, as a threshold for the number of connections, the mean performs favourably against either inter-quartile.

**Table 3:** Precision analysis of the guilt-by-association algorithm

			Threshold for number of connections (TC)			
			
Threshold for % disease genes among interactors (TD)			Q1 TC = 1	Q2 TC = 4	Q3 TC = 13	Mean TC = 12
Q1	TD = 12.8	N Captured	1,943	1,391	638	683
		% Known	73.3	75.0	76.5	76.4

Q2	TD = 28.6	N Captured	1,024	563	195	219
		% Known	74.8	78.9	85.1	84.9

Q3	TD = 50.0	N Captured	251	118	16	19
		% Known	70.5	67.8	75.0	78.9

Mean	TD = 35.0	N Captured	748	409	109	127
		% Known	73.4	76.3	84.4	84.2

The optimality of various location parameters to be used as thresholds in the guilt-by-association algorithm was explored by computing the proportion of known (% Known) disease associated genes from the total number of captured genes (N Captured). The analysis was performed using only the 1,445 genes (out of the initial 6,151) with known disease phenotype as the set of truly disease causing, and with the remaining 4,706 declared as disease associated. The three inter-quartiles (Q1: 25^th ^percentile; Q2: 50^th ^percentile or median; and Q3: 75^th ^percentile) plus the mean were used as thresholds.

However, the infeasibility of directly computing performance measures associated with a given algorithm in the absence of negative examples should be acknowledged. That is, although one can be relatively sure that certain genes are associated with a disease, it is not possible to ensure that a set of genes is not involved in any disease. In other words: Absence of evidence is not evidence of absence. On the other extreme, some of the genes annotated as disease associated by OMIM could also be false positives. In these situations, partially supervised learning algorithms have been proposed to address this issue and in the context of identifying disease genes [14].

Nevertheless, a literature survey revealed that 44 of the 112 candidate genes [see Additional file 2] have been previously associated with polymorphisms or differential gene expression leading to a modified risk of disease. A further 10 genes exist within chromosomal regions associated with disease. The remaining 58 genes have no obvious association to disease in any system. The 39% rate of disease association determined here is much higher (hypergeometric P = 7.5 × 10^-16^) than the 14% predicted by OMIM across the genome, with 2,549 genes defined as the basis of heritable disease out of the 18,091 total.

### Clusters of disease among candidate genes

**Figure 5:**
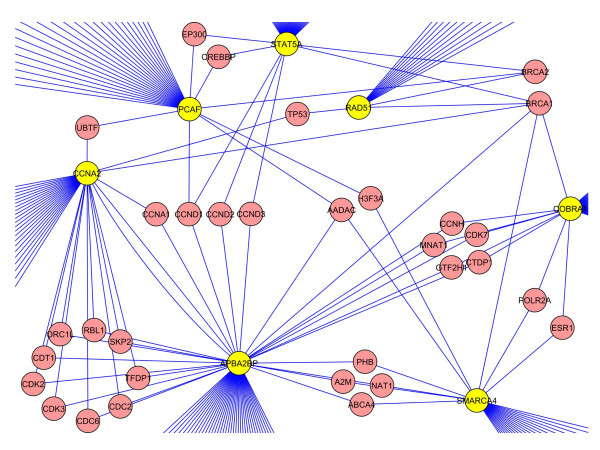
**Guilt-by-association network analysis on breast cancer**. A cluster of 7 non-disease associated genes (yellow) each interacting with BRCA1 and/or BRCA2.

In order to determine what diseases these genes might impact, we explored the gene networks spanned by the members of our guilt-by-association list, alone and in combination with their interactors. Based on the disease associations shown [see Additional file 2], each cluster was examined for a common disease. In this fashion, we identified two clusters of genes that impact on either breast or gastric cancer. Figure 5 depicts the Cytoscape [25] representation of the breast cancer cluster where seven of our guilt-by-association genes (APBA2BP, CCNA2, COBRA1, PCAF, RAD51, SMARCA4 and STAT5A) were linked to the well characterized human breast cancer susceptibility genes, BRCA1 and BRCA2. Although none of these genes are annotated as disease causing in OMIM, five have been previously associated with the development of breast cancer, for example, alleles of RAD51 are epistatic with alleles of BRCA2. However, CCNA2 is only mentioned in a very small number of reports on breast cancer and APBA2BP is not a well studied gene.

For the case of gastric cancer, another cluster of seven genes (AKT3, KRAS, MAP2K4, PIK3CB, PLCB1, PIK3R5 and PPP3R2) was identified. Four of these genes have been previously associated with gastrointestinal disease while AKT3, PIK3CB and PIK3R5 have not, although the differential expression of AKT3 in gastric cancer is well defined [see Additional file 2]. We suggest these previously non-associated genes are strong candidates for further study into the basis of these diseases and are potential prognostic markers.

## Conclusion

Data mining approaches have allowed us to gain an insight into the complex relationships existing between gene expression, disease association, network connectivity and tissue specificity. We have identified elevated rates of expression and network connectivity among broadly expressed genes, and among disease-associated tissue-specific genes.

In particular, when exploring the relationship between tissue specificity and disease association, we found this relationship most interesting. While there is a moderate positive relationship between the number of tissues in which a gene is expressed and the proportion of disease genes, we show that this relationship is reversed when only considering genes for which interactions have been reported. We present this phenomenon as an example of the well-reported Simpson's Paradox. To a great extent, the inclusion of number of interactions as a threshold parameter in our guilt-by-association algorithm obviates the need to also include tissue specificity.

However, it should also be acknowledged that probability values associated with testing the null hypothesis of a given PCC not being statistically different from zero were computed assuming asymptotic normality and as such are prone to inaccuracies. With this in mind, we focussed on combining discrete parameters such as number of connections and the association to disease-associated genes to identify a group of genes, not previously confirmed as disease causing, that are involved in interactions with disease causing genes. The nature of these newly identified interactions could range from epistatic interactions (i.e., the action of one gene is suppressed by another such as the case of RAD51 and BRCA1) to physical gene-gene interactions to correlated co-expression. Based on bibliographical validation and network re-construction we have identified several candidate genes that may impact the development of cancer and hypothesize that many other members of this list will ultimately be confirmed as modifiers of various genetic diseases.

Finally, it should be noted that while new algorithms are being proposed in the literature on a rather frantic pace, the task of comprehensively comparing algorithms could be unattainable if not futile. Instead, we claim that our conservative thresholds for predicting disease association is justified because using thresholds of known disease genes increases our likelihood of success given any estimation process is going to have a degree of false positives. We acknowledge the list does not exhaust all possible disease genes but merely gives researchers the best short list for further study.

## Abbreviations

HK: housekeeping; MPSS: massively parallel signature sequencing; NS: non-specific; PCC: Pearson correlation coefficient; TS: tissue-specific; DIS: genes in our dataset that are disease-associated according to OMIM as of September 19, 2007; NDIS: genes in our dataset that are non-disease-associated genes also according to OMIM; INT: genes in our dataset for which interactions have been reported; NINT: genes in our dataset for which interactions have not been reported.

## Competing interests

The authors declare that they have no competing interests.

## Authors' contributions

AR conceived the study, carried out the data mining approaches and drafted the manuscript. AI directed the design and coordination of the biological/immunological relevance of the results and drafted the manuscript. BD participated in the coordination of the whole study and drafted the manuscript. All authors read and approved the final manuscript.

## Supplementary Material

Additional file 1 Additional Table 1: The set of 15,050 genes. List of 15,050 genes included in the analyses. For each gene, the number of tissues (out of 32) in which the gene is being expressed, its average expression, disease association and connectivity structure is provided. Click here for file

Additional file 2 Additional Table 2: Set of 112 guilt-by-association genes. List of 112 genes not associated with disease according to OMIM yet with high connectivity with disease-associated genes. For each gene, the proportion of disease genes among connectors and polymorphism or differential expression associated with disease along with the relevant literature reference is provided. Click here for file
