# Supplementary material for: Mining tissue specificity, gene connectivity and disease association to reveal a set of genes that modify the action of disease causing genes
Source: BioData Min. 2008 Sep 19;1:8. doi: 10.1186/1756-0381-1-8 (PMC2556670; doi:10.1186/1756-0381-1-8)
Supplement: Additional file 2 — Additional Table 2: Set of 112 guilt-by-association genes. List of 112 genes not associated with disease according to OMIM yet with high connectivity with disease-associated genes. For each gene, the proportion of disease genes among connectors and polymorphism or differential expression associated with disease along with the relevant literature reference is provided. [file 1756-0381-1-8-S2.doc]

**Mining tissue specificity, gene connectivity and disease association to reveal a set of genes that modify the action of disease causing genes**

Antonio Reverter, Aaron Ingham, Brian P. Dalrymple

Computational and Systems Biology, CSIRO Livestock Industries, Queensland Bioscience Precinct, 306 Carmody Road, St. Lucia, Brisbane, Queensland 4067, Australia.

**Additional Table 2: Guilt-by-Association list: 112 genes not associated with disease according to OMIM yet with high connectivity with disease-associated genes**

| **Gene** | **Alias** | **Proportion of disease genes among connectors** | **Polymorphism or differential expression associated with disease** | **Reported in disease QTL** |
| --- | --- | --- | --- | --- |
|
|  |
| ACAA2 | DSAEC | 0.851 | No | No |
| ACVR2A | ACTRII | 0.923 | Gastrointestinal cancer ^45^ | No |
| ADHFE1 | FLJ32430 | 1.000 | No | No |
| AKR1B10 | ARL-1 | 0.812 | No | No |
| AKR1D1 | SRD5B1 | 0.928 | Hepatitis ^32^ | No |
| AKT3 | PKBG | 0.794 | No | No |
| APBA2BP | XB51 | 0.870 | No | No |
| APEX1 | APE | 0.842 | Cancer ^10^ | No |
| BCDO2 |  | 0.823 | No | No |
| BIRC2 | cIAP1 | 0.846 | Cancer ^3^ | No |
| CCNA2 |  | 0.916 | Cancer ^50^ | No |
| CCR5 |  | 1.000 | Protection against HIV infection ^22^ | No |
| CDT1 |  | 0.763 | Cancer ^26^ | No |
| COBRA1 |  | 0.800 | Cancer ^39^ | No |
| COPS5 | JAB1 | 0.760 | Cancer ^13^ | No |
| CXXC5 | HSPC195 | 0.928 | No | No |
| DARS |  | 0.782 | No | No |
| DTYMK |  | 0.823 | No | No |
| DVL2 |  | 0.805 | No | No |
| E2F5 |  | 0.928 | Cancer ^47^ | No |
| ENTPD4 | LALP70 | 0.784 | No | No |
| ESCO1 | ESO1 | 0.756 | No | No |
| FLJ40504 |  | 0.941 | No | No |
| FRS2 | SNT1 | 0.933 | No | No |
| GADD45A |  | 0.846 | Cancer ^54^ | No |
| GGTL3 | D20S101 | 0.869 | No | No |
| GGTLA1 | GGT-REL | 0.869 | No | Developmental ^14^ |
| GLI1 |  | 0.814 | Cancer ^9^ | No |
| GMPPA |  | 0.846 | No | No |
| GMPPB |  | 0.846 | No | No |
| GNA12 |  | 0.869 | No | Cancer ^23^ |
| GNA13 |  | 0.888 | No | No |
| GNG12 |  | 0.857 | No | No |
| GPX5 |  | 0.823 | No | No |
| GTF2B |  | 0.760 | No | Cancer ^52^ |
| GTF2E1 |  | 0.764 | No | No |
| GTPBP4 | CRFG | 0.772 | Kidney Disease ^31^ | No |
| HNRPAB |  | 0.937 | No | No |
| HSD17B12 |  | 0.812 | No | No |
| HTATIP | TIP60 | 0.857 | Cancer ^18^ | No |
| IDI2 |  | 0.791 | No | No |
| IL10RB |  | 0.900 | Severity of organ rejection ^36^ | Malaria susceptibility ^28^ |
| IL22RA1 |  | 0.896 | No | No |
| IL23A |  | 0.906 | Psoriasis ^6^ | No |
| ITGB5 |  | 0.961 | Cancer ^29^ | No |
| ITGB8 |  | 1.000 | No | Spinocerebellar ataxia ^11^ |
| KHDRBS1 | SAM68 | 0.800 | No | No |
| KNG1 |  | 0.950 | Diabetes ^51^ | No |
| KRAS |  | 0.833 | Cancer ^42^ | No |
| LDHAL6A |  | 0.923 | No | No |
| LDHAL6B |  | 0.923 | No | No |
| MAP2K4 | MKK4 | 0.760 | Cancer ^7^ | No |
| NAALAD2 |  | 0.875 | No | No |
| NCOA2 |  | 0.833 | Leukaemia ^35^ | No |
| NCOR1 |  | 0.812 | No | Cancer ^15^ |
| NMNAT1 |  | 0.842 | No | No |
| NMNAT2 |  | 0.842 | No | No |
| NMNAT3 |  | 0.842 | No | Alzheimers ^37^ |
| NT5C1B | AIRP | 0.760 | No | No |
| NUDT12 |  | 0.789 | No | No |
| NUP205 |  | 0.863 | No | No |
| NUP93 |  | 0.863 | No | No |
| NUPL2 |  | 0.863 | No | No |
| OSMR |  | 0.900 | Cancer ^44^ | No |
| PCAF |  | 0.935 | Cancer ^46^ | No |
| PCK2 | PEPCK | 0.933 | Viral Resistance ^34^ | No |
| PDE10A |  | 0.878 | Psychosis ^21^ | No |
| PDE1C |  | 0.878 | Pulmonary Disease ^49^ | No |
| PDE2A |  | 0.878 | No | No |
| PDE3A |  | 0.880 | Heart failure ^12^ | No |
| PDE7B |  | 0.878 | Anaemia ^53^ | No |
| PDE8B |  | 0.878 | No | No |
| PDGFD |  | 0.842 | No | No |
| PDHX |  | 0.857 | Acidosis ^1^ | No |
| PIK3CB |  | 0.802 | No | No |
| PIK3R5 |  | 0.763 | No | No |
| PLCB1 |  | 0.780 | No | Alagille syndrome ^48^ |
| PMAIP1 | NOXA | 0.882 | No | No |
| POLA2 |  | 0.888 | No | No |
| POLD3 |  | 0.863 | No | No |
| POLD4 |  | 0.863 | No | No |
| POLR1A | RPA1 | 0.787 | Cancer ^41^ | No |
| POLR1B |  | 0.829 | No | No |
| POLR1C |  | 0.822 | No | No |
| POLR1D |  | 0.818 | No | No |
| POLR3C |  | 0.896 | No | No |
| POLR3K |  | 0.896 | No | No |
| PPP3R2 |  | 0.888 | IBD ^17^ | No |
| PRDX6 |  | 0.882 | No | No |
| PRKAB2 |  | 0.785 | No | No |
| RAD51 |  | 0.928 | Cancer Susceptibility^24^ | No |
| RAE1 |  | 0.869 | No | No |
| RCOR1 | COREST | 0.785 | Cancer ^8^ | No |
| RDH13 |  | 0.812 | No | No |
| RHOB |  | 0.818 | Osteoarthritis ^38^ | No |
| RIPK1 |  | 0.875 | No | No |
| SDC3 |  | 0.806 | Obesity ^20^ | No |
| SDCBP |  | 1.000 | No | No |
| SH3GLB1 |  | 0.780 | No | Cancer ^2^ |
| SHC3 |  | 0.862 | Nicotine dependence ^33^ | No |
| SMARCA4 |  | 0.931 | Cancer ^40^ | No |
| SMARCE1 | BAF57 | 0.857 | Cancer ^30^ | No |
| SOCS2 |  | 0.909 | Diabetes ^27^ | No |
| SOCS6 |  | 0.877 | Tubercolosis relapse ^43^ | No |
| STAT5A |  | 0.878 | Fat content of milk ^5^ | No |
| TAF13 |  | 0.757 | No | No |
| TRIP4 |  | 0.846 | No | No |
| VEGFC |  | 0.842 | Acceptance of kidney transplant ^19^ | No |
| WNT9B |  | 0.941 | Cleft palate ^25^ | No |
| YWHAQ |  | 0.888 | No | No |
| ZBTB16 | PLZF | 0.913 | Cancer | Cancer ^4^ |
| ZNRD1 |  | 0.896 | Progression of HIV ^16^ | No |

**References**

1. Aral, B. *et al.* Mutations in PDX1, the human lipoyl-containing component X of the pyruvate dehydrogenase-complex gene on chromosome 11p1, in congenital lactic acidosis. *Am J Hum Genet* **61**, 1318-26 (1997).

2. Balakrishnan, A. *et al.* Quantitative microsatellite analysis to delineate the commonly deleted region 1p22.3 in mantle cell lymphomas. *Genes Chromosomes Cancer* **45**, 883-92 (2006).

3. Bashyam, M. D. *et al.* Array-based comparative genomic hybridization identifies localized DNA amplifications and homozygous deletions in pancreatic cancer. *Neoplasia* **7**, 556-62 (2005).

4. Baysal, B. E. *et al.* A high-resolution STS, EST, and gene-based physical map of the hereditary paraganglioma region on chromosome 11q23. *Genomics* **44**, 214-21 (1997).

5. Brym, P., Kaminski, S., & Rusc, A. New SSCP polymorphism within bovine STAT5A gene and its associations with milk performance traits in Black-and-White and Jersey cattle. *J Appl Genet* **45**, 445-52 (2004).

6. Cargill, M. *et al.* A large-scale genetic association study confirms IL12B and leads to the identification of IL23R as psoriasis-risk genes. *Am J Hum Genet*  **80**, 273-90 (2007).

7. Chae, K. S., Ryu, B. K., Lee, M. G., Byun, D. S., & Chi, S. G. Expression and mutation analyses of MKK4, a candidate tumour suppressor gene encoded by chromosome 17p, in human gastric adenocarcinoma. *Eur J Cancer* **38**, 2048-57 (2002).

8. Coulson, J. M. Transcriptional regulation: cancer, neurons and the REST. *Curr Biol* **15**, R665-8 (2005).

9. Dahmane, N., Lee, J., Robins, P., Heller, P., & Ruiz, i. Altaba A. Activation of the transcription factor Gli1 and the Sonic hedgehog signalling pathway in skin tumours. *Nature* **389**, 876-81 (1997).

10. De Ruyck, K. *et al.* Polymorphisms in base-excision repair and nucleotide-excision repair genes in relation to lung cancer risk. *Mutat Res* **631**, 101-10 (2007).

11. Delplanque, J. *et al.* Slowly progressive spinocerebellar ataxia with extrapyramidal signs and mild cognitive impairment (SCA21). *Cerebellum* 1-5 (2007).

12. Ding, B. *et al.* Functional role of phosphodiesterase 3 in cardiomyocyte apoptosis: implication in heart failure. *Circulation* **111**, 2469-76 (2005).

13. Dong, Y. *et al.* Prognostic significance of Jab1 expression in laryngeal squamous cell carcinomas. *Clin Cancer Res* **11**, 259-66 (2005).

14. Edelmann, L., Pandita, R. K., & Morrow, B. E. Low-copy repeats mediate the common 3-Mb deletion in patients with velo-cardio-facial syndrome. *Am J Hum Genet* **64**, 1076-86 (1999).

15. Fabris, S. *et al.* Molecular and transcriptional characterization of the novel 17p11.2-p12 amplicon in multiple myeloma. *Genes Chromosomes Cancer* (2007).

16. Fellay, J. *et al.* A whole-genome association study of major determinants for host control of HIV-1. *Science* **317**, 944-7 (2007).

17. Franke, A. *et al.* Systematic association mapping identifies NELL1 as a novel IBD disease gene. *PLoS ONE* **2**, e691 (2007).

18. Gorrini, C. *et al.* Tip60 is a haplo-insufficient tumour suppressor required for an oncogene-induced DNA damage response. *Nature* **448**, 1063-7 (2007).

19. Gunesacar, R. *et al.* VEGF 936 C/T gene polymorphism in renal transplant recipients: association of the T allele with good graft outcome. *Hum Immunol* **68**, 599-602 (2007).

20. Ha, E. *et al.* Positive association of obesity with single nucleotide polymorphisms of syndecan 3 in the Korean population. *J Clin Endocrinol Metab* **91**, 5095-9 (2006).

21. Hebb, A. L. & Robertson, H. A. Role of phosphodiesterases in neurological and psychiatric disease. *Curr Opin Pharmacol* **7**, 86-92 (2007).

22. Hill, C. M. & Littman, D. R. Natural resistance to HIV? *Nature* **382**, 668-9 (1996).

23. Imreh, S. *et al.* Nonrandom loss of human chromosome 3 fragments from mouse-human microcell hybrids following progressive growth in SCID mice. *Genes Chromosomes Cancer* **11**, 237-45 (1994).

24. Jakubowska, A. *et al.* The RAD51 135 G>C polymorphism modifies breast cancer and ovarian cancer risk in Polish BRCA1 mutation carriers. *Cancer Epidemiol Biomarkers Prev* **16**, 270-5 (2007).

25. Juriloff, D. M., Harris, M. J., McMahon, A. P., Carroll, T. J., & Lidral, A. C. Wnt9b is the mutated gene involved in multifactorial nonsyndromic cleft lip with or without cleft palate in A/WySn mice, as confirmed by a genetic complementation test. *Birth Defects Res A Clin Mol Teratol* **76**, 574-9 (2006).

26. Karakaidos, P. *et al.* Overexpression of the replication licensing regulators hCdt1 and hCdc6 characterizes a subset of non-small-cell lung carcinomas: synergistic effect with mutant p53 on tumor growth and chromosomal instability--evidence of E2F-1 transcriptional control over hCdt1. *Am J Pathol* **165**, 1351-65 (2004).

27. Kato, H. *et al.* Association of single-nucleotide polymorphisms in the suppressor of cytokine signaling 2 (SOCS2) gene with type 2 diabetes in the Japanese. *Genomics* **87**, 446-58 (2006).

28. Khor, C. C. *et al.* Positive replication and linkage disequilibrium mapping of the chromosome 21q22.1 malaria susceptibility locus. *Genes Immun* (2007).

29. Kim, T. M. *et al.* Determination of genes related to gastrointestinal tract origin cancer cells using a cDNA microarray. *Clin Cancer Res* **11**, 79-86 (2005).

30. Kiskinis, E., Garcia-Pedrero, J. M., Villaronga, M. A., Parker, M. G., & Belandia, B. Identification of BAF57 mutations in human breast cancer cell lines. *Breast Cancer Res Treat* **98**, 191-8 (2006).

31. Laping, N. J., Olson, B. A., & Zhu, Y. Identification of a novel nuclear guanosine triphosphate-binding protein differentially expressed in renal disease. *J Am Soc Nephrol* **12**, 883-90 (2001).

32. Lemonde, H. A. *et al.* Mutations in SRD5B1 (AKR1D1), the gene encoding delta(4)-3-oxosteroid 5beta-reductase, in hepatitis and liver failure in infancy. *Gut* **52**, 1494-9 (2003).

33. Li, M. D. *et al.* Linkage and association studies in African- and Caucasian-American populations demonstrate that SHC3 is a novel susceptibility locus for nicotine dependence. *Mol Psychiatry* **12**, 462-73 (2007).

34. Li, S., Zadworny, D., Aggrey, S. E., & Kuhnlein, U. Mitochondrial PEPCK: a highly polymorphic gene with alleles co-selected with Marek's disease resistance in chickens. *Anim Genet* **29**, 395-7 (1998).

35. Liang, J., Prouty, L., Williams, B. J., Dayton, M. A., & Blanchard, K. L. Acute mixed lineage leukemia with an inv(8)(p11q13) resulting in fusion of the genes for MOZ and TIF2. *Blood* **92**, 2118-22 (1998).

36. Lin, M. T. *et al.* Genetic variation in the IL-10 pathway modulates severity of acute graft-versus-host disease following hematopoietic cell transplantation: synergism between IL-10 genotype of patient and IL-10 receptor beta genotype of donor. *Blood* **106**, 3995-4001 (2005).

37. Liu, F. *et al.* A genomewide screen for late-onset Alzheimer disease in a genetically isolated Dutch population.  *Am J Hum Genet* **81**, 17-31 (2007).

38. Mahr, S. *et al.* Cis- and trans-acting gene regulation is associated with osteoarthritis. *Am J Hum Genet* **78**, 793-803 (2006).

39. McChesney, P. A. *et al.* Cofactor of BRCA1: a novel transcription factor regulator in upper gastrointestinal adenocarcinomas. *Cancer Res* **66**, 1346-53 (2006).

40. Medina, P. P. *et al.* Genetic and epigenetic screening for gene alterations of the chromatin-remodeling factor, SMARCA4/BRG1, in lung tumors. *Genes Chromosomes Cancer* **41**, 170-7 (2004).

41. Michiels, S. *et al.* Polymorphism discovery in 62 DNA repair genes and haplotype associations with risks for lung and head and neck cancers. *Carcinogenesis* **28**, 1731-9 (2007).

42. Minamoto, T., Mai, M., & Ronai, Z. K-ras mutation: early detection in molecular diagnosis and risk assessment of colorectal, pancreas, and lung cancers--a review. *Cancer Detect Prev* **24**, 1-12 (2000).

43. Mistry, R. *et al.* Gene-expression patterns in whole blood identify subjects at risk for recurrent tuberculosis.  *J Infect Dis* **195**, 357-65 (2007).

44. Ng, G. *et al.* Gain and overexpression of the oncostatin M receptor occur frequently in cervical squamous cell carcinoma and are associated with adverse clinical outcome. *J Pathol* **212**, 325-34 (2007).

45. Olaru, A. *et al.* Loss of heterozygosity and mutational analyses of the ACTRII gene locus in human colorectal tumors. *Lab Invest* **83**, 1867-71 (2003).

46. Ozdag, H. *et al.* Differential expression of selected histone modifier genes in human solid cancers. *BMC Genomics* **7**, 90 (2006).

47. Polanowska, J. *et al.* Human E2F5 gene is oncogenic in primary rodent cells and is amplified in human breast tumors. *Genes Chromosomes Cancer* **28**, 126-30 (2000).

48. Pollet, N. *et al.* Construction of an integrated physical and gene map of human chromosome 20p12 providing candidate genes for Alagille syndrome. *Genomics*  **42**, 489-98 (1997).

49. Schermuly, R. T. *et al.* Phosphodiesterase 1 upregulation in pulmonary arterial hypertension: target for reverse-remodeling therapy. *Circulation* **115**, 2331-9 (2007).

50. Schick, V. *et al.* Mutational and expression analysis of CDK1, cyclinA2 and cyclinB1 in epilepsy-associated glioneuronal lesions. *Neuropathol Appl Neurobiol* **33**, 152-62 (2007).

51. Vionnet, N. *et al.* Analysis of 14 candidate genes for diabetic nephropathy on chromosome 3q in European populations: strongest evidence for association with a variant in the promoter region of the adiponectin gene. *Diabetes* **55**, 3166-74 (2006).

52. Walker, G. J. *et al.* Deletion mapping suggests that the 1p22 melanoma susceptibility gene is a tumor suppressor localized to a 9-Mb interval. *Genes Chromosomes Cancer* **41**, 56-64 (2004).

53. Wyszynski, D. F. *et al.* Polymorphisms near a chromosome 6q QTL area are associated with modulation of fetal hemoglobin levels in sickle cell anemia. *Cell Mol Biol (Noisy-le-grand)* **50**, 23-33 (2004).

54. Yamasawa, K., Nio, Y., Dong, M., Yamaguchi, K., & Itakura, M. Clinicopathological significance of abnormalities in Gadd45 expression and its relationship to p53 in human pancreatic cancer. *Clin Cancer Res* **8**, 2563-9 (2002).
